# Forgoing Exchange Transfusion in Neonatal Hyperbilirubinemia: A Single-Center Retrospective Cohort Study

**DOI:** 10.7759/cureus.56749

**Published:** 2024-03-22

**Authors:** Nourelhouda Ouerradi, Anass Ayyad, Sahar Messaoudi, Rim Amrani

**Affiliations:** 1 Neonatology and Neonatal Resuscitation, Mohammed First University Faculty of Medicine, Oujda, MAR

**Keywords:** jaundice, kernicterus, neonatal hyperbilirubinemia, exchange transfusion, intensive phototherapy

## Abstract

Introduction: Unconjugated hyperbilirubinemia is part of the everyday life of the neonatal period as it reflects the adaptation of the metabolism of bilirubin. The neonatal hyperbilirubinemia usually resolves spontaneously, but it can also be the cause of an acute or chronic encephalopathy known as kernicterus. Regardless of the cause, the goal of therapy is to prevent this neurotoxicity while not causing undue harm. Phototherapy and, if it is unsuccessful, exchange transfusion (ECT) remain the primary treatment modalities used to keep the maximal total serum bilirubin (TSB) below pathologic levels.

Materials and methods: This is a descriptive retrospective cohort study of 69 live neonates hospitalized in the Department of Neonatology and Neonatal Resuscitation of Mohammed VI University Hospital with unconjugated hyperbilirubinemia requiring ECT and treated with intensive phototherapy instead, spanning five years from March 2016 to March 2021. We aim to demonstrate the effectiveness of phototherapy in achieving prolonged reduction of bilirubin levels and the prevention of neurological complications and to compare our results with those in the literature.

Results: The use of intensive phototherapy in the treatment of neonatal unconjugated hyperbilirubinemia is very effective in lowering total serum bilirubin when its level is in the range of exchange transfusion, and it has succeeded in preventing the neurological complications of severe hyperbilirubinemia.

Conclusion: Through this study, it can be seen that phototherapy is an efficacious, simpler, and less hazardous alternative to exchange transfusion in achieving a sustained reduction of bilirubin levels and preventing neurological complications.

## Introduction

Unconjugated hyperbilirubinemia is a common occurrence during the neonatal period. Although neonatal hyperbilirubinemia typically resolves on its own, it can potentially lead to an acute or chronic condition known as kernicterus. This complication persists worldwide and results in severe and irreversible neurological consequences. The exact incidence of kernicterus is unknown; however, most recent data from the United Kingdom and Canada suggests that kernicterus occurs at a rate of 1-2 in 100,000 live births. Clinical manifestations may range from subtle, with or without auditory neuropathy, to more severe presentations constituting the classic picture of kernicterus. Regardless of the etiology, the primary objective of treatment is to prevent neurotoxicity while minimizing potential harm.

While exchange transfusion (ECT) has proven effective in reducing dangerously high serum bilirubin levels, the introduction of phototherapy, a simpler and less hazardous procedure, appears to diminish the necessity for exchange transfusion in managing severe neonatal hyperbilirubinemia.

## Materials and methods

This study is a comprehensive retrospective cohort analysis involving 69 neonates who were admitted to the Department of Neonatology and Neonatal Resuscitation of Mohammed VI University Hospital, Oujda, Morocco. These neonates exhibited unconjugated hyperbilirubinemia, necessitating exchange transfusion (ECT) as per the guidelines outlined by the American Academy of Pediatrics (AAP) and the French Society of Neonatology. Instead of pursuing ECT, these neonates underwent intensive phototherapy over a period of five years, spanning from March 2016 to March 2021.

The phototherapy employed in this study utilized super light-emitting diode (LED) bed technology, equipped with 17 super LEDs and an arc reflector to optimize the treatment's efficacy. The wavelength of the light ranged from 480 to 510 nm, with a light intensity between 20 and 30 µW/cm^2^/nm. Neonates were exposed unclothed with covered eyes and genitals during the sessions. The phototherapy sessions commenced immediately after determining capillary plasma bilirubin levels, repeated at 12-hour/six-hour intervals, with lights turned off during blood sample collection. Neonates were temporarily removed from the LED beds during feeding intervals occurring every three hours. Phototherapy was only discontinued after achieving bilirubin stabilization at a safe level.

All cases underwent a thorough clinical evaluation, with a particular emphasis on the neurological examination conducted by the department team. Blood tests were consistently conducted by the same laboratory under identical conditions. Before statistical analysis, data was assessed for normality and homogeneity variances. Descriptive statistics included categorical variables presented as numbers and percentages (N and %), and continuous variables were described using mean and standard deviation (mean±SD).

Statistical analysis involved the chi-square McNemar test for comparing categorical variables and the paired t-test for continuous variables. A two-tailed p-value of <0.05 was considered statistically significant. The data analysis was executed using the Statistical Package for Social Sciences (SPSS) 26.0 software (IBM SPSS Statistics, Armonk, NY). The primary objective of this study is to demonstrate the efficacy of phototherapy, a simpler and less hazardous alternative, in achieving the sustained reduction of bilirubin levels and preventing neurological complications. Our findings were compared with existing literature to contribute to a comprehensive understanding of the benefits of phototherapy in neonatal hyperbilirubinemia management.

## Results

We studied 69 infants who were born at more than 35 gestational weeks with hyperbilirubinemia requiring exchange transfusion according to the French Society of Neonatology guidelines adapted from the subcommittee on hyperbilirubinemia, held by the American Academy of Pediatrics on the management of hyperbilirubinemia in the newborn infant at 35 or more weeks of gestation. The prevalence of neonates with hyperbilirubinemia requiring ECT in our department was 4.8%. The majority of our patients were full-term newborns (88%). We found a negligible male predominance; the sex ratio was almost 1. According to birth weight, 43% of the newborns were appropriate for gestational age, 4% were large for gestational age, and 3% were less than 2 kg. The median age at the start of the phototherapy treatment was two days, with a minimum of one day and a maximum of four days.

In our series, the leading cause of neonatal jaundice appears to be ABO blood group incompatibility, followed by rhesus incompatibility. Additionally, 33 patients showed dual etiologies, frequently a combination of incompatibility with an infection, accounting for 81% of these cases, or co-occurrence of the ABO blood group and rhesus incompatibility, making up 15.2% of the cases.

At the beginning of the treatment, 83% of the neonates were asymptomatic, 15% had moderate neurological symptoms, and 3% or two patients were hospitalized with kernicterus already; at the end of the treatment, none of the initially asymptomatic neonates developed neurological signs, the patients who had moderate signs at the beginning became asymptomatic, and the two patients already with kernicterus kept neurological sequelae. The chi-square McNemar test tested the results obtained for the neurological state before and after sessions; the p-value was 0.002 (<5%) (Table 1). Therefore, phototherapy is significantly effective in preventing the worsening of neurological signs and progression to kernicterus.

**Table 1:** The chi-square McNemar test used to test the results (the effect of phototherapy on the neurological state of the patients) *Binomial distribution used

	Value	Exact significance (two-sided)
McNemar test		0.002*
Number of valid cases	69	

The evolution of the mean total serum bilirubin (TSB) in our series decreased (from 454.95±137.05 to 185.05±67.85); no rebound was described, whatever the etiology. The paired sample t-test was used to test the results for changes in total serum bilirubin, with a p-value of less than 0.001 well below 5% (Table 2). It is therefore concluded that intensive phototherapy is very effective in lowering total serum bilirubin when its level is in the range of exchange transfusion, whatever its etiology.

**Table 2:** The paired sample test used to evaluate the total serum bilirubin results before and after phototherapy sessions

	95% confidence interval of the difference	Significance (two-tailed)
Pair: bilirubin level 0 to bilirubin level end	235.9396-303.9293	0.000

## Discussion

Hyperbilirubinemia impacts a significant proportion of neonates, affecting up to 85% of full-term infants (≥37 weeks of gestational age) and 80% of premature newborns [1-3]. Although severe hyperbilirubinemia is rare in term infants, its occurrence is linked to permanent neurodevelopmental delay and the development of kernicterus [2]. Kernicterus, preventable through various measures including phototherapy, intravenous immunoglobulins (IVIg), or exchange transfusion (ECT), can occur in 20% of infants with a total serum bilirubin (TSB) exceeding 30 mg/dL [4,5].

ECT was not followed when the bilirubin was in the exchange zone because donating blood has not yet become a reflex for Moroccans. In 2022, 339,579 donations were collected nationwide. The percentage of donations to the population was 0.92%, and the percentage of donations to the population by region was as follows: 1.2% in the east (the region to which our center belongs) according to the Centre National de Transfusion Sanguine et d'Hématologie (CNTSH) on the situation of blood donation in Morocco. We are still far from the minimum recommendations of the World Health Organization (WHO); the current average in developed countries is four times higher than that of Morocco, at 3.9%.

In addition, we often found ourselves in situations where we needed the mother's blood grouping for a safe transfusion, but she could not be reached, either because she lived far away or because her state of health or means did not allow it, and it would be unethical not to treat the patient with the available means.

ECT, considered more efficient in reducing bilirubin levels, is employed when maximal phototherapy and/or IVIg prove unsuccessful or in cases of excessive hemolysis [6-8]. However, ECT is a complex procedure associated with several complications. It necessitates the presence of at least three individuals at the bedside, involving a credentialed clinician operating exchange cycles, a second clinician/nurse monitoring and handling the infant, and a third individual with a clinical background documenting the events. The procedure's intricacies include considerations for the volume and rate of blood withdrawal/infusion, catheter placements, and continuous vital signs monitoring.

Adverse events related to ECT encompass a spectrum of complications such as death, sepsis, electrolyte imbalances, air embolism, and thrombotic events, emphasizing its role as a last line of defense in developed countries [9-13]. The immediate and marked fall in bilirubin levels during ECT is transient, with a rapid rebound influencing subsequent decisions in managing neonatal severe hyperbilirubinemia [14].

The present study reveals that intensive light-emitting diode (LED) phototherapy is significantly effective in lowering total serum bilirubin within the range of exchange transfusion, irrespective of its etiology, as per the AAP and French Society of Neonatology guidelines. Importantly, LED phototherapy demonstrates the potential to prevent severe neurological complications, notably kernicterus, thereby reducing the reliance on exchange transfusion and mitigating associated adverse events. Earlier studies indicate phototherapy's effectiveness in preventing a TSB greater than 20 mg/dL, with an 84% reduction in the need for exchange transfusion [15].

While phototherapy has short-term adverse effects such as diarrhea, intestinal hypermotility, temperature instability, and interference with maternal-infant bonding [13,16], these were not observed in our series. These side effects, which are manageable and sometimes avoidable, pale in comparison to those associated with exchange transfusion (ECT).

The study's findings align with the results from international studies [14,17-23], underscoring the lack of mortality as a key advantage of phototherapy. Phototherapy emerges as the therapy of choice in preventing and treating neonatal jaundice, significantly reducing the need for exchange transfusion by two-thirds in full-term infants and nearly 80% in preterm infants. While phototherapy acts more gradually than ECT, resulting in a small and inconsequential rebound, its efficacy in achieving prolonged reduction of bilirubin levels, especially in non-hemolytic hyperbilirubinemia, is evident. Importantly, it proves effective even in cases of hemolytic jaundice.

While the study provides valuable insights into the efficacy of intensive LED phototherapy as an alternative to exchange transfusion (ECT) in managing severe neonatal hyperbilirubinemia, there are several limitations to consider.

The study is conducted at a specific neonatal department in Mohammed VI University Hospital, Oujda, Morocco. A single-center study may not fully capture the diversity of patient populations and clinical practices seen across different institutions. Additionally, the retrospective design might introduce selection bias and limit the control over data collection.

The study involves a cohort of 69 neonates. While this sample size may be appropriate for a single-center study, it could still limit the generalizability of the findings. Larger, multicenter studies would provide a more robust understanding of the effectiveness of LED phototherapy.

While the study suggests that LED phototherapy is effective in reducing bilirubin levels and preventing complications, a direct comparison with ECT is not provided. To draw more robust conclusions about the superiority of LED phototherapy, a randomized controlled trial comparing the two interventions would be beneficial.

The study focuses on the reduction of bilirubin levels and the prevention of neurological complications, particularly kernicterus. Other relevant outcomes, such as long-term neurodevelopmental follow-up or the rates of readmission, could enhance the comprehensiveness of the study.

The study uses a specific type of LED phototherapy technology. The effectiveness of LED phototherapy may vary depending on the specific technology, intensity, and duration of exposure. Additionally, the availability and affordability of such technology may differ across healthcare settings.

Addressing these limitations and considering them in the interpretation of results will contribute to a more accurate and nuanced understanding of the role of LED phototherapy in managing neonatal hyperbilirubinemia.

## Conclusions

Exchange transfusion, though effective for severe unconjugated hyperbilirubinemia, carries risks escalating with repeated use due to the rebound phenomenon. This poses challenges and raises concerns about the overall safety of exchange transfusion.

Advanced phototherapy technology, featuring efficient lamps, offers a compelling opportunity to reassess the conventional reliance on exchange transfusion. Particularly in cases where repeated exchanges may be necessary, harnessing the efficacy of phototherapy holds promise. As we shift toward innovative strategies in neonatal care, integrating efficient phototherapy lamps offers a noninvasive and safer alternative to exchange transfusion, highlighting its potential to mitigate risks and prevent neurological complications. Further research and clinical exploration are essential to solidify phototherapy's role as a cornerstone in the comprehensive management of neonatal hyperbilirubinemia.
